# Capsule-Like Smart Aggregate with Pre-Determined Frequency Range for Impedance-Based Stress Monitoring

**DOI:** 10.3390/s23010434

**Published:** 2022-12-30

**Authors:** Quang-Quang Pham, Quoc-Bao Ta, Jeong-Tae Kim

**Affiliations:** Department of Ocean Engineering, Pukyong National University, 45 Yongso-ro, Nam-gu, Busan 48513, Republic of Korea

**Keywords:** capsule-like, smart aggregate, PZT sensor, impedance response, concrete damage, compressive loading, stress monitoring

## Abstract

In this article, a new capsule-like smart aggregate (CSA) is developed and verified for impedance-based stress monitoring in a pre-determined frequency range of less than 100 kHz. The pros and cons of the existing smart aggregate models are discussed to define the requirement for the improved CSA model. The conceptual design and the impedance measurement model of the capsule-like smart aggregate (CSA) are demonstrated for concrete damage monitoring. In the model, the interaction between the CSA and the monitored structure is considered as the 2-degrees of freedom (2-DOF) impedance system. The mechanical and impedance responses of the CSA are described for two conditions: during concrete strength development and under compressive loadings. Next, the prototype of the CSA is designed for impedance-based monitoring in concrete structures. The local dynamic properties of the CSA are numerically simulated to pre-determine the sensitive frequency bands of the impedance signals. Numerical and experimental impedance analyses are performed to investigate the sensitivity of the CSA under compressive loadings. The changes in the impedance signals of the CSA induced by the compressive loadings are analyzed to assess the effect of loading directions on the performance of the CSA. Correlations between statistical impedance features and compressive stresses are also made to examine the feasibility of the CSA for stress quantification.

## 1. Introduction

Structural health monitoring (SHM) at an early stage plays the essential role of ensuring structural integrity. It also helps enhance the efficiency of the structural maintenance process. In recent decades, several local monitoring techniques have been developed for concrete structures [1,2]. Among those techniques, impedance-based damage monitoring has been considered as a promising method [3,4]. The theoretical background of impedance-based monitoring was proposed by Liang et al. [5] and subsequently followed by many researchers [6,7]. The prominent characteristic of the method is to obtain electromechanical impedance responses of high resonant frequency ranges by employing a coupling interaction between a piezoelectric sensor (e.g., lead zirconate titanate, PZT) and a monitored structure. The variation in the impedance signals sensitively represents the change in the monitored structure. With this basic concept, the method has been widely used to diagnose various damage types (e.g., cracks, loose connections, corrosion, and prestress force loss) in different structures [8,9,10].

Conventionally, surface-bonded PZTs have been widely applied for damage monitoring in concrete structures, such as concrete surface crack detection [8,11] and concrete strength development during the curing process [12]. PZT patches were directly mounted on the surface of the target structure using adhesives such as instant glue or epoxy resin. However, impedance features measured by the surface-bonded PZT sensors could be affected by various factors. These factors were related to PZT’s characteristics (e.g., fragility, instability under temperature, and humidity changes [13]) and the quality of the bonding layer between the PZT patch and the host structures [14]. To overcome the shortcomings of the surface-bonded PZTs, coated-PZTs (protected by covering layers of epoxy resin) were embedded into concrete structures during the concrete casting process [15,16]. The coated-PZTs were also installed into concrete blocks to form smart aggregates (SAs) [17]. The embedded PZT sensor could measure changes in impedance signatures induced by changes in internal stresses near the sensor location. Many researchers have implemented the embedded PZT sensors for concrete strength monitoring [17,18,19], inner concrete damage monitoring [20,21], and quantitative concrete damage assessment [22]. The effects of sensor orientation and temperature variation on impedance signals have also been investigated for embedded PZT sensors [23].

Despite extensive research efforts, there are important issues regarding the SA technique that must be solved to guarantee its applicability to real structures. The first issue is to pre-determine the frequency ranges of impedance responses that are sensitive to local structural damage before being installed into concrete structures. For impedance monitoring using embedded PZT sensors, most previous studies utilized the trial-and-error method to search sensitive frequency ranges [23]. Some studies pre-determined proper frequency bands using analytical and numerical methods [21,22]. The second issue is to measure impedance signals in low-frequency ranges of less than 100 kHz, which is required for the wireless impedance sensor node [24]. In recent studies on the embedded PZT sensors, most of the impedance responses were measured in frequency ranges larger than 100 kHz [18,21,22,25]. Therefore, there exists a research motivation to ensure the outstanding characteristics of SA sensors by improving the limitations on measurable frequency ranges.

In this study, a new capsule-like smart aggregate (CSA) is developed for the impedance-based stress monitoring of a concrete structure in a pre-determined frequency range of less than 100 kHz. The pros and cons of the existing smart aggregate models are discussed to define the requirement for the improved CSA sensor. The conceptual design and the impedance measurement model of the CSA sensor are demonstrated for concrete damage monitoring. The interaction between the CSA and the monitored structure is modeled as the 2-degrees of freedom (2-DOF) impedance system. The mechanical and impedance responses of the CSA during concrete curing and under compressive loading conditions are calculated. Next, the prototype of the CSA is designed for impedance-based monitoring in concrete structures. The local dynamic properties of the CSA are numerically simulated to obtain impedance signatures in pre-determined frequency bands. Numerical and experimental impedance analyses are performed for the CSA to investigate its sensitivity under compressive loadings. The variation in the impedance signatures of the CSA induced by the compressive loadings are analyzed to assess the effect of loading directions on the performance of the CSA. The correlations between statistical impedance features and compressive stresses are also examined to verify the feasibility of the CSA for stress quantification.

This paper is arranged as follows: (i) Section 2 presents the theoretical model of the CSA in the concrete structure; (ii) Section 3 presents the numerical impedance analysis of the CSA in the concrete structure; (iii) Section 4 presents the experimental impedance analysis of the CSA under compression; and (iv) Section 5 draws the findings and conclusions. The novelty and research significance of this study are described as follows: (i) a new capsule-like smart aggregate (CSA) with a pre-determined frequency range of less than 100 kHz for impedance measurement is developed; (ii) the mechanical and impedance responses of the CSA during concrete strength development and under compressive loading are calculated; (iii) the feasibility of the CSA for stress quantification is verified via numerical and experimental analyses; (iv) the effect of the various loading directions on the CSA’s impedance responses is investigated to help localize the sensor in the concrete structure, in order to enhance the impedance-based stress monitoring.

## 2. Theoretical Model of CSA in Concrete Structure

### 2.1. Impedance Model of CSA

**Figure 1:**
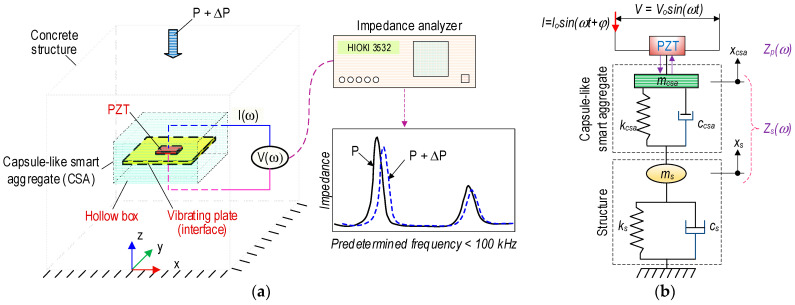
Conceptual model of CSA in concrete structure under compression. (**a**) CSA-embedded concrete structure. (**b**) 2-DOF impedance model.

A capsule-like smart aggregate model was newly developed to overcome the aforementioned limitations of the smart aggregate models. The CSA was developed based on the concept of the PZT interface technique proposed by Huynh et al. [10]. With the PZT interface, impedance signals could be measured in frequency ranges of less than 100 kHz. Moreover, the desired frequency range could be pre-determined by specifying the material and geometric parameters of the PZT interface. As illustrated in Figure 1a, a CSA embedded in a concrete structure measures the impedance responses induced by external forces. The CSA consists of a PZT patch, an interface (i.e., vibrating plate), and a capsule (i.e., hollow box). The interface is a flexible metal plate with fixed ends, and a PZT patch is surface-mounted in the middle of the plate. The PZT interface is intentionally designed to allow its flexural vibration responses according to the piezoelectric deformation of the PZT sensor. Moreover, the sensitive frequency ranges of the impedance signals can be pre-determined by specifying the material, geometrical, and boundary conditions of the PZT interface [10,26]. The capsule plays the role of a PZT-embedded sensor for local impedance monitoring. The hollow box protects the PZT interface during concrete curing. The capsule device allows the PZT interface to produce as-designed impedance signals of the concrete structure under compression.

Once the compressive force changes (i.e., Δ*P*), it leads to the corresponding variation in structural properties (e.g., stiffness, mass, and damping) of the CSA-embedded zone. When an electrical voltage *V*(*ω*) is applied to the PZT patch (see Figure 1a), it vibrates to transfer a mechanical strain to the CSA and a local area of the target structure via the inverse piezoelectric effect. Simultaneously, the structural responses (e.g., mechanical stresses) are conversely transferred to the PZT patch to generate corresponding voltage signals via the direct piezoelectric effect. For the practical application, an electromechanical impedance (EMI) response *Z*(*ω*) is acquired as the ratio between the input voltage *V*(*ω*) and the output current *I*(*ω*). The EMI response is dependent not only on the structural mechanical (SM) impedance of the PZT patch, but also on that of the CSA–host structure [5]:

(1) $$Z(\omega )=\frac{V\left(\omega \right)}{I\left(\omega \right)}={\left\{i\omega a_{p}\left[{\hat{\varepsilon }}_{33}^{T}-\frac{1}{Z_{p}(\omega )/Z_{s}\left(\omega \right)+1}d_{31}^{2}{\hat{Y}}_{11}^{E}\right]\right\}}^{-1}$$

where *i* is the imaginary unit; ω signifies the excitation frequency; *a_p_* is the geometric parameters constant of the piezoelectric patch. The terms ${\hat{\varepsilon }}_{33}^{T}$, *d*_31_*,* and ${\hat{Y}}_{11}^{E}$ are, respectively, the complex dielectric constant at zero stress, the piezoelectric constant in one direction at zero stress, and the complex Young’s modulus of the PZT patch at zero electric fields. Moreover, *Z_p_*(*ω*) is the SM impedance of the PZT (i.e., *Z_p_*(*ω*) = ${\hat{Y}}_{11}^{E}a_{p}/\left(i\omega \right)$. *Z_s_*(*ω*) is the coupling SM impedance of the CSA and the host structure. Thus, the measured impedance response *Z*(*ω*) represents the mechanical properties of the CSA-monitored structure. The SM impedance of the PZT-embedded CSA is assumed to keep constant before and after damage occurrence. Thus, changes in the mechanical properties of the target structure can be determined by quantifying the variation of *Z*(*ω*).

As shown in Figure 1b, a two-degrees of freedom (2-DOF) impedance model represents the coupling system of the CSA and the embedded structure [10]. One DOF is for the motion of the structure, and the other is for the motion of the CSA. The parameters *m_csa_*, *c_csa_*, and *k_csa_* are the mass, damping coefficient, and stiffness of the CSA. Then, *m_s_*, *c_s_*, and *k_s_* are the corresponding parameters of the concrete structure. The coupled SM impedance *Z_s_*(*ω*) of the CSA and the host structure at the driving point of the PZT are computed as follows:

(2) $$Z_{s}\left(\omega \right)=\frac{K_{11}\left(\omega \right)K_{22}\left(\omega \right)-K_{12}^{2}\left(\omega \right)}{i\omega K_{22}\left(\omega \right)}$$

where *K*_11_, *K*_12_, and *K*_22_ are the dynamic stiffness components [10], which contain the structural parameters of the CSA and the target structure. It can be noted that the stiffness coefficients rely on the structural properties of the monitored structure and the CSA. Once the PZT’s features are constant, any external effects (e.g., a transformation of the concrete medium, applied force alteration, or concrete defect) would impact the SM impedance responses of the CSA–target structure. Thus, any changes in the inspected structure could be monitored using the PZT-embedded CSA.

### 2.2. Mechanical and Impedance Behaviors of CSA

**Figure 2:**
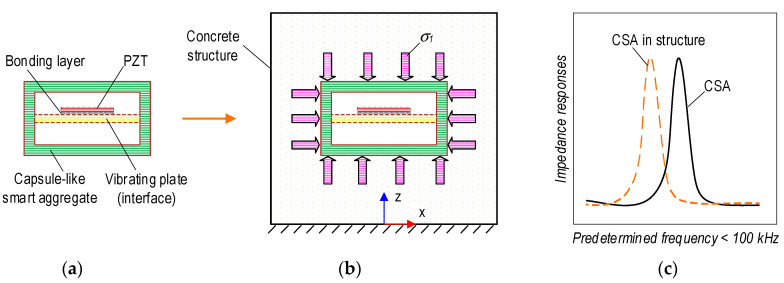
Behavior of CSA in concrete structure during concrete strength development. (**a**) CSA sensor. (**b**) CSA embedded in structure. (**c**) Impedance responses.

Figure 2 shows the CSA’s mechanical and impedance responses in a concrete structure during concrete strength development. It is assumed that no stress acts on the CSA during its fabrication (see Figure 2a). The CSA is placed in the concrete structure before concrete casting. Then, the CSA is compressed by stress *σ*_1_ induced by the hardening process of the concrete material (see Figure 2b). As shown in Figure 2c, the impedance response of the CSA could be changed, since the pre-stress *σ*_1_ could lead to compression on the vibrating plate of the CSA sensor.

**Figure 3:**
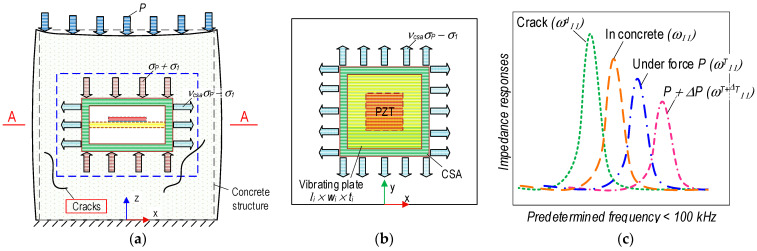
Behavior of CSA in concrete structure under compressive loading. (**a**) CSA in structure. (**b**) PZT interface in A-A. (**c**) Impedance signals.

Figure 3 presents the mechanical and impedance behaviors of the CSA in the concrete structure under compression. Due to the compressive force *P*, stress–strain responses occur in the CSA embedded in the concrete structure (see Figure 3a). The corresponding impedance responses are acquired from the PZT-mounted vibrating plate in the CSA (see Figure 3b). The CSA’s surfaces are subjected to compression stress, *σ_P_*, along the vertical direction (i.e., z-direction) and under tension stress, *ν_csa_σ_P_*, induced by Poisson’s effect (with *ν_csa_* being Poisson’s ratio of the CSA’s material) for other surfaces. As a result, the compressive stress acting on the CSA is increased along the z-direction *(σ*_1_ + *σ_P_*), but it is decreased for other directions (i.e., x and y axes) (*ν_csa_σ_P_* − *σ*_1_). Furthermore, the stress acting on the concrete structure along the x and y directions is determined by a value *ν_s_σ_P_* (with *ν_s_* being Poisson’s ratio of the concrete material).

As zoomed in Figure 3b, the PZT-mounted vibrating plate has a size of length × width × thickness = *l_i_* × *w_i_* × *t_i_*_,_ of the CSA embedded in the concrete structure. The vibrating plate is considered as a clamped rectangular plate in the CSA. It undergoes a tension force, which is induced by the tensile stress (*ν_csa_σ_P_* − *σ*_1_) (see Figure 3b). As shown in Figure 3c, the impedance responses of the CSA can be acquired as corresponding to the working conditions of the concrete structure. The impedance resonant frequency of the CSA in the concrete after the curing process is *ω*_11_. The CSA’s frequency will be changed to *ω^T^*_11_ if the tensile stress *ν_csa_σ_P_* induced by applied force *P* is applied on the vibrating plate. The change in applied forces correspondingly leads to the change in the tensile stress applied to the vibrating plate, thus, causing the shift in the CSA’s impedance responses *ω^T+^*^Δ*T*^_11_. The tensile stresses applied to the vibrating plate would be suddenly released as local cracks are developed close to the CSA. This phenomenon suggests that the concrete medium surrounding the CSA is transformed, resulting in sudden changes in the impedance responses of the CSA sensor [20,21]. The CSA’s frequency in the crack state is *ω^d^*_11_. Ideally, the CSA’s frequency in the free state will be returned when the concrete structure is a failure.

## 3. Numerical Impedance Analysis of CSA in Concrete Structure

### 3.1. Design of CSA

#### 3.1.1. Prototype of CSA Sensor

**Figure 4:**
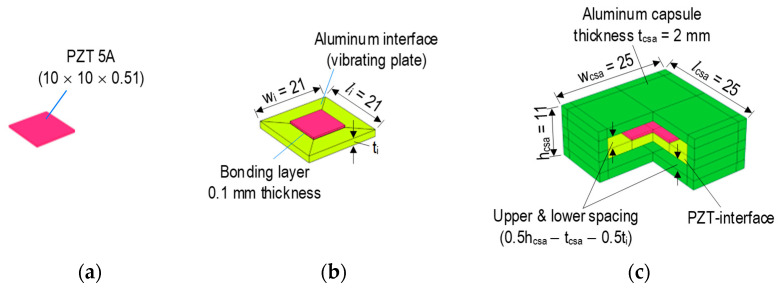
Prototype design of PZT-embedded capsule-like smart aggregate (dimension in mm). (**a**) PZT patch. (**b**) PZT interface. (**c**) CSA.

Figure 4 presents a newly developed prototype of a PZT-embedded CSA sensor. A thin PZT patch (10 × 10 × 0.51 mm) (see Figure 4a) was connected with electric wires. The PZT was surface-bonded on an aluminum vibrating plate (length × width × thickness = *l_i_* × *w_i_* × *t_i_*) via a thin bonding layer (about 0.1 mm thickness) to form a PZT interface (see Figure 4b). In the current design, the length and the width of the interface were selected to be the same (*l_i_* × *w_i_* = 21 × 21 mm). The interface’s thickness was varied to investigate the effect of its geometry on the sensitive impedance frequency range studied in the following sections. The PZT interface was embedded in the center of a hollow aluminum box (thickness 2 mm) to form a capsule-like smart aggregate (see Figure 4c). The hollow box had a size of (length × width × height = *l_csa_* × *w_csa_* × *h_csa_ =* 25 × 25 × 11 mm). The PZT interface and the top and bottom plates of the box were spaced at a distance of 0.5*h_csa_ − t_csa_* − 0.5*t_i_ =* 3.5 − 0.5*t_i_*. The spacing helped ensure electric insulation and made the interface easy to vibrate. After fabrication, the CSA was embedded in the target concrete structures for impedance monitoring.

#### 3.1.2. Local Dynamic Characteristics of PZT-Mounted Interface

As described previously, the CSA consists of the PZT-mounted interface (vibrating plate) and the capsule box. Compared to the flexural aluminum interface, the cover box is relatively rigid to protect the capsule. Thus, the dynamic characteristics of the CSA can be represented by the modal properties of the PZT-mounted interface. Previous studies demonstrated that impedance frequencies sensitive to local damage coincided with the resonant responses of the PZT-mounted interfaces [10,26]. Therefore, in this study, the local dynamic characteristics of the PZT interface (see Figure 4b) were analyzed to pre-determine the resonant impedance peaks of the CSA sensor.

**Figure 5:**
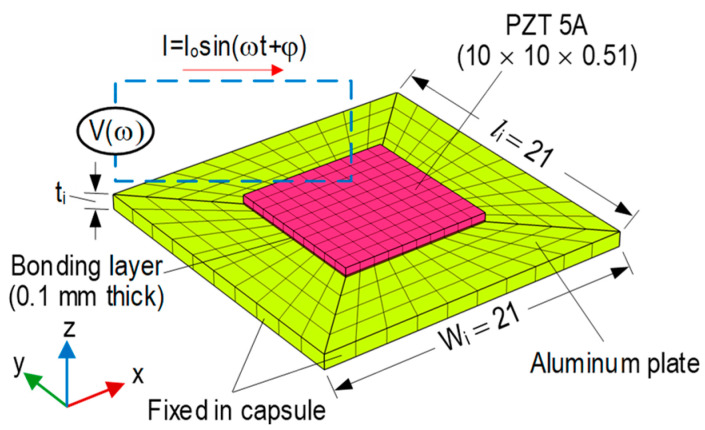
FE model of PZT-mounted interface in CSA (dimension in mm).

Figure 5 shows a finite element (FE) model of the PZT interface in the CSA sensor using COMSOL Multiphysics. The model was simulated to determine the sensitive impedance frequency range. To investigate the effect of the interface’s geometry on the resonant impedance frequency range, the thickness of the vibrating plate was considered as 1 mm (Interface 1), 1.5 mm (Interface 2), and 2.0 mm (Interface 3). The 0.1 mm thick bonding layer was simulated as the contact between the PZT sensor and the vibrating plate. The material properties of the aluminum, bonding layer, and PZT patch of the CSA sensor are listed in Table 1 [10,26]. The PZT interface was simulated with 460 elements, including 100 elements for the bonding layer, 100 for the PZT sensor, and 260 for the aluminum plate. The quadratic hexahedron elements were used for the PZT interface. All edges of the aluminum vibrating plate were assigned as fixed boundary conditions.

**Figure 6:**
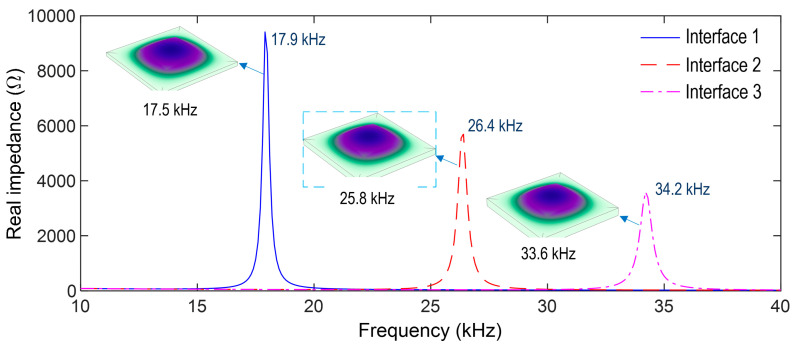
Impedance peaks and corresponding eigen-modes of three PZT interfaces.

An impedance analysis was performed for the PZT interface. The top surface of the PZT sensor was excited by a harmonic voltage of 1 V, while the bottom surface was applied as the ground electrode. As shown in Figure 6, the impedance responses of the three PZT interfaces (i.e., Interfaces 1–3) were numerically simulated in the frequency range of 10–40 kHz. Each interface had a resonant impedance peak: 17.9 kHz for Interface 1, 26.4 kHz for Interface 2, and 34.2 kHz for Interface 3. It is noted that the impedance frequency proportionally increased with the increase in the interface thickness, thus, suggesting that the impedance responses obtained from the PZT interface could be controlled via the interface’s geometric parameters. An eigenvalue analysis was performed on the three PZT interfaces to identify the eigen-modes corresponding to the impedance peaks. As shown in Figure 6, a plate-bending mode was estimated in the range of 10–40 kHz for each interface: 17.5 kHz for Interface 1, 25.8 kHz for Interface 2, and 33.6 kHz for Interface 3. It is observed that the sensitive frequency range of the CSA’s impedance signatures can be pre-determined via the eigenvalue analysis. Interface 2 (thickness 1.5 mm) was selected for the CSA prototype.

### 3.2. Numerical Impedance Responses of CSA under Compression

#### 3.2.1. FE Analysis of CSA under Compression

**Figure 7:**
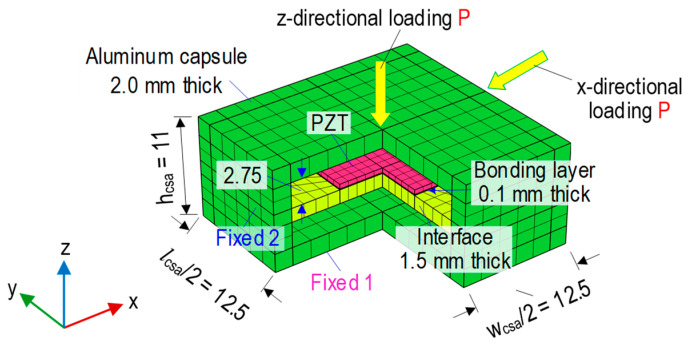
FE model of CSA under compression (dimension in mm).

Figure 7 shows an FE model of the CSA sensor under compression, which was built to investigate the sensitivity of the CSA sensor for impedance monitoring in the pre-determined frequency range. The thickness of 1.5 mm (Interface 2) was selected for the vibrating plate. The material characteristics of the CSA were assumed to be linear elastic during the compressive loadings. The CSA’s FE model consists of 968 elements, including 100 elements for the bonding layer, 100 for the PZT, 260 for the aluminum interface (i.e., vibrating plate), and 508 for the aluminum box. The quadratic hexahedron elements were used for all components. A uniform compression force *P* was applied on the top surface of the CSA sensor, and the fixed boundary condition was assigned to the bottom one.

**Figure 8:**
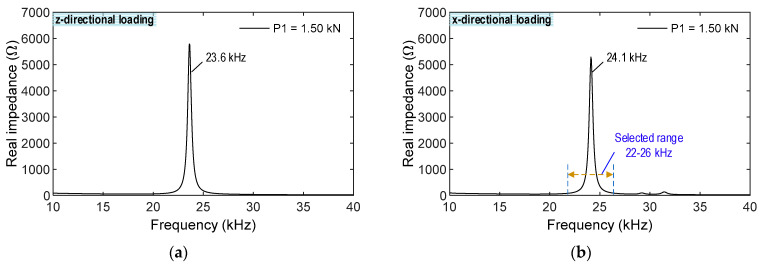
CSA’s impedance signals in frequency range of 10–40 kHz for intact case. (**a**) z-directional loading. (**b**) x-directional loading.

**Figure 9:**
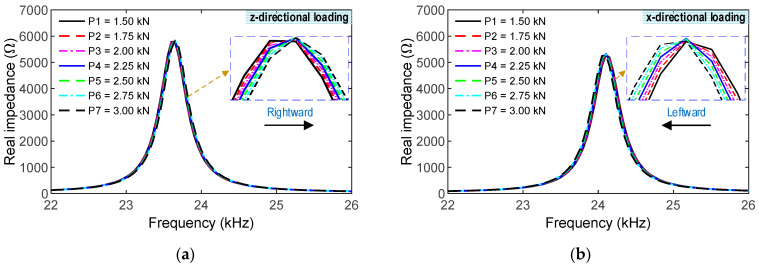
CSA’s impedance signals in a selected range of 22–26 kHz under loading cases. (**a**) z-directional loading. (**b**) x-directional loading.

To investigate the effect of the different loading directions, the CSA was assigned with compressive forces in both the z- and x-directions (see Figure 7). Seven loading scenarios (P1-P7) of the compressive forces were simulated to acquire the impedance signatures of the CSA (see Figure 8 and Figure 9). A sequence of applied forces was listed as follows: P1 (1.5 kN), P2 (1.75 kN), P3 (2.0 kN), P4 (2.25 kN), P5 (2.5 kN), P6 (2.75 kN), and P7 (3.0 kN). The CSA’s impedance signals were analyzed in the frequency range of 10–40 kHz. Figure 8 shows the impedance responses of the CSA under the z- and x-directional loadings in the range of 10–40 kHz under loading P1. For the CSA under the z-directional loading, there was only one prominent impedance peak at 23.6 kHz in the simulated range, thus, confirming the pre-determined sensitive frequency range of the CSA using the vibrating plate. For the CSA under the x-directional loading, the main impedance peak was at 24.1 kHz, which was slightly larger than for the z-directional loading. It is assumed that P1 (1.5 kN) loading is the intact case.

As shown in Figure 9, the frequency range of 22–26 kHz, which contains the main peak, was investigated to quantify the changes in impedance signatures induced by the changes in compression forces. For the z-directional loading, the impedance signals were shifted to the right under loading cases P1–P7, and they were relatively varied with respect to the applied force variations. For the x-directional loading, the impedance signatures were shifted to the left under loading cases P1–P7, and they were relatively changed, corresponding to the applied force alterations. The variations in the impedance signals of the CSA under x-directional loading were slightly higher than those under z-directional loading.

#### 3.2.2. Numerical Impedance Features of CSA under Compression

To quantify the changes in impedance signals, the RMSD (root mean square deviation) index and the CCD (correlation coefficient deviation) index are commonly used as damage indicators for the characterization of structural damage.

The RMSD index is computed as follows [6]:

(3) $$\mathrm{RMSD}\left(Z,Z^{*}\right)=\sqrt{\left(\sum_{i=1}^{N}{\left[Z^{*}(\omega_{i})-Z(\omega_{i})\right]}^{2}\right)/\sum_{i=1}^{N}{\left[Z(\omega_{i})\right]}^{2}}$$

where *Z*(*ω_i_*) and Z*(*ω_i_*) are the impedance signals in the intact and damaged states of the structure at *i*th frequency, respectively, and *N* denotes the number of frequency sampling points in the sweep.

The CCD index is determined as follows [7]:

(4) CCD=1−1σzσz*EReZi−ReZ¯ReZi*−ReZ¯*

where *σ_Z_* denotes the standard deviation values of impedance signals before and after the damage event; *E*(.) is the expectation operation; Re(*Z_i_*) is the real components of the impedances of the *i*th frequency in the intact and damaged cases; and ReZ¯ is the mean values of impedance signatures before and after damage. The asterisk (*) signifies the damaged case.

**Figure 10:**
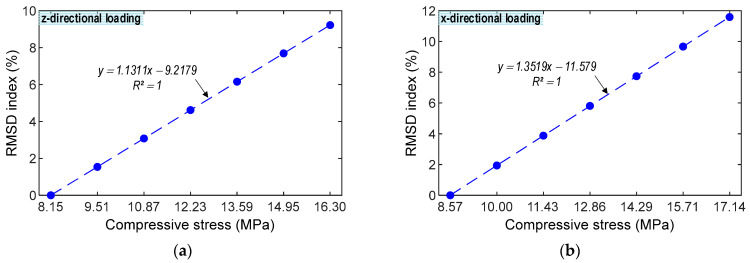
Numerical correlation estimation between RMSD index and compressive stress. (**a**) z-directional loading. (**b**) x-directional loading.

**Figure 11:**
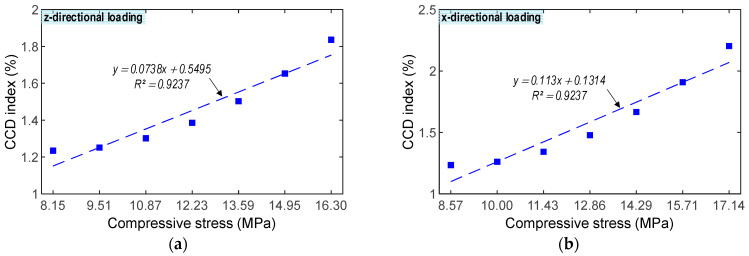
Numerical correlation estimation between CCD index and compressive stress. (**a**) z-directional loading. (**b**) x-directional loading.

The correlations between impedance features and stresses under z- and x-directional loadings were examined, as shown in Figure 10 and Figure 11. Figure 10 illustrates the relationship between the RMSD index and the stress value. The RMSD magnitudes were linearly increased, corresponding to the increase in the forces P1–P7. The RMSD indices quantified from the impedance signals of the CSA under the x-directional loading were slightly higher than those under the z-directional loading. The correlation coefficient *R^2^* reached 1 for the CSA under both z- and x-directional loadings, suggesting the strong relation between the RMSD index quantified from the variation in the numerical impedance signatures and the stress value.

Figure 11 demonstrates the relationship between the CCD index and stress. The CCD values were insignificantly increased under applied forces. The CCD indices quantified from the impedance signals of the CSA under the x-directional loadings were slightly higher than those under the z-directional loadings. The correlation coefficient *R*^2^ reached 0.9237 for the CSA under both z- and x-directional loadings, suggesting the significant relation between the CCD index quantified from the variation in the numerical impedance signatures and the compressive stress.

## 4. Experimental Impedance Analysis of CSA under Compression

### 4.1. Fabrication of CSA Prototype

**Figure 12:**
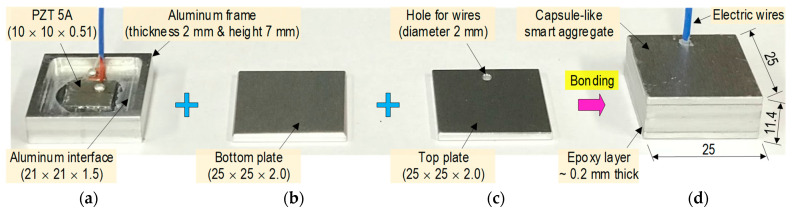
Fabrication of CSA prototype (dimension in mm). (**a**) PZT interface. (**b**) Bottom cover. (**c**) Top cover. (**d**) CSA.

The CSA prototype was fabricated by assembling its components. Figure 12 shows the fabrication process of the CSA sensor that was selected based on the numerical analysis described previously. As shown in Figure 12, a PZT 5A patch (10 × 10 × 0.51 mm) was connected to electric wires, and it was surface-mounted on an aluminum vibrating plate (21 × 21 × 1.5 mm). The vibrating plate was positioned at the center of an aluminum wall frame (thickness of 2 mm and height of 7 mm) to form a PZT-mounted interface (see Figure 12a). Then, the top and bottom plates (25 × 25 × 2 mm) covered the PZT interface by epoxy-bonding to form a CSA sensor (see Figure 12d). The top plate was designed with a hole (diameter of 2 mm) for passing the electric wires (see Figure 12c). An epoxy resin [27] was used for the bonding layers (about 0.2 mm thickness). A CSA sample (25 × 25 × 11.4 mm) was fabricated by bonding all parts using super glue and epoxy (see Figure 12d). The material properties used for the CSA are listed in Table 1.

**Figure 13:**
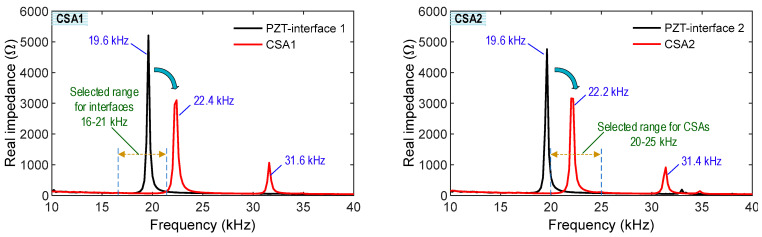
Impedance signals of CSAs during fabrications.

As shown in Figure 13, the impedance responses corresponding to the fabrication steps were experimentally measured for two CSA samples (CSA 1 and CSA 2). The impedance responses were examined for two stages: (1) a PZT interface (see Figure 12a) and (2) a complete CSA (see Figure 12d). At the stage of the PZT interface, only one impedance peak was measured in the frequency range of 10–40 kHz (151 points). Both PZT interfaces 1 and 2 had a peak impedance at 19.6 kHz. At the stage of the CSA, two impedance peaks were measured in the frequency of 10–40 kHz (151 points). CSA 1 had two impedance peaks at 22.4 kHz and 31.6 kHz, shifting from the peak impedance from the PZT interface 1. CSA 2 had two impedance peaks at 22.2 kHz and 31.4 kHz, shifting from the peak impedance from the PZT interface 2.

The impedance peaks of the PZT interfaces were reduced in magnitude and increased in frequency after the fabrication of the CSA sensors (see Figure 13). Furthermore, minor peaks at 31.6 kHz for CSA 1 and 31.4 kHz for CSA 2 were observed in the CSA stage. These observations could be induced by the increase in the CSA’s whole structural stiffness and the change in the boundary condition induced by the fabrication. As compared to the numerical impedance analysis (see Figure 8), the experimental impedance responses of the CSA samples had similar patterns in the pre-determined frequency range (10–40 kHz). Furthermore, the experimental impedance signatures of CSAs 1–2 were almost identical to each other, suggesting the reliability of the CSA’s fabrication process and the CSA sensors for impedance monitoring.

### 4.2. Test Setup for CSA under Compression

**Figure 14:**
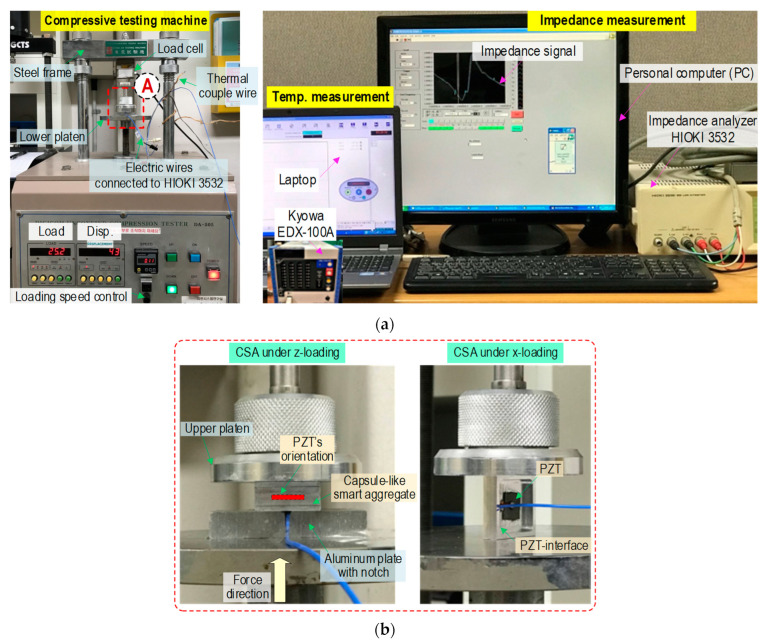
Test setup for CSAs under compression. (**a**) Compressive testing machine and impedance measuring system. (**b**) Detail of A: CSAs under compression.

As shown in Figure 14, a series of tests were conducted on the CSA to investigate its performance of impedance monitoring under compressive loadings. The CSA samples were examined under both z- and x-directional loadings. Two fabricated CSAs were used to test under the z-directional loadings, and two PZT interfaces (which remove the top and bottom cover plates of the CSAs) were used to examine under the x-directional loadings. It is noted that the covers were opened to monitor potential buckling in the PZT interface due to the in-plane compression loads (see Figure 14b). As marked in Figure 13, the frequency range of 20–25 kHz was selected for the two CSA samples under the z-directional loadings. Moreover, the frequency range of 16–21 kHz was selected for the PZT interfaces under the x-directional loadings. As shown in Figure 14b, a CSA sensor was positioned for the z-directional loadings in the compressive testing machine and an aluminum plate (10 mm thickness). The compression forces were applied to the CSA with a loading speed of 0.05 mm/min and controlled by using a load cell. The two CSA samples (namely CSA 1 and CSA 2) were used for impedance monitoring under the z-directional loading. As also shown in Figure 14b, a PZT interface without the top/bottom covers was directly positioned for the x-directional loadings. The two PZT interfaces (namely CSA 3 and CSA 4) were used for impedance monitoring under the x-directional loadings.

For the loadings, the applied forces were increased from 0 kN to 3.0 kN with a force interval of 0.25 kN. The impedance signals of all sensors (i.e., CSAs 1–2 and CSAs 3–4) were suddenly shifted to the left and achieved a stable condition at the applied force of 1.5 kN. To set the fixed boundary conditions for the CSAs, the compression force of 1.5 kN was set as the baseline. The variations in the impedance signals of sensors under compression could be observed with seven remaining loading cases from P1 (1.5 kN) to P7 (3.0 kN) with an interval of 0.25 kN. For the impedance measurement, a wired impedance analyzer HIOKI 3532 (see Figure 14a) was used to stimulate a 1 V harmonic voltage and record the impedance signals. For the z-directional loading, the impedance signals of CSA 1 and CSA 2 were measured in the frequency range of 20–25 kHz (101 points). For the x-directional loading, the impedance signals of CSA 3 and CSA 4 were measured in the frequency range of 16–21 kHz (101 points). During the experiment, the laboratory temperature was controlled at around 20 °C (measured via Kyowa EDX-100A) to minimize the effects of temperature variation on the impedance features. For each loading case, four ensembles of impedance responses were obtained to determine the control threshold UCL [28] and the error bars. Furthermore, four measured ensembles also helped to reduce the effects of noise (e.g., electrical noise) on the impedance features, which were computed based on the variations in the impedance signatures. The UCL was used as an alert for damage occurrence [28], and the error bars were employed to check the stability of the impedance signals.

### 4.3. Experimental Impedance Responses of CSA under Compression

#### 4.3.1. Experimental Impedance Responses of CSAs

**Figure 15:**
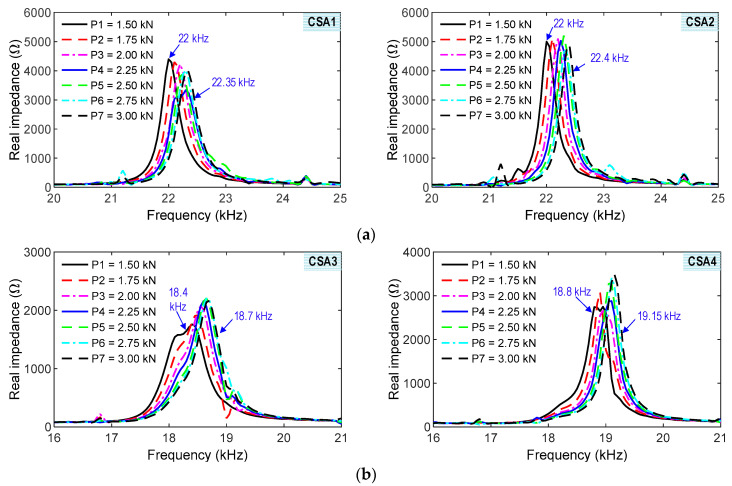
Experimental impedance signals of CSAs under compression. (**a**) z-directional loading. (**b**) x-directional loading.

Figure 15a shows the measured impedance responses of CSA 1 and CSA 2 under the seven z-directional loading cases (i.e., P1–P7). The two CSA samples (CSA 1 and CSA 2) had similar trends of impedance signals induced by the loadings. The peak frequencies of the impedance signals were increased as the compressive forces were increased. The tendency of the experimental impedance responses was consistent with the numerical impedance analyses (see Figure 9a). The alteration in the impedance responses of CSA 2 was higher than those of CSA 1. The difference could be caused by the effects of the sensor fabrication, the bonding condition, and the compressive testing setup.

Figure 15b shows the measured impedance responses of CSA 3 and CSA 4 under the seven x-directional loading cases (i.e., P1–P7). The peak frequencies of the impedance signals were increased as the compressive forces were increased. The variation tendency of the experimental impedance responses was inconsistent with the numerical impedance analyses (see Figure 9b), which could be induced by the stiffening effect due to fixed boundary conditions [29]. The alteration in the impedance responses of CSA 3 was relatively higher than those of CSA 4. It can be observed that there was a different form in the impedance signals of CSAs 3–4 under loading P1 by comparing with other cases. This phenomenon could be induced by the effects of the testing setup and fixed conditions of the CSAs on the compressive machine. The CSAs 1–2 under the z-directional loadings had relatively higher sensitivity to the compressive forces than the CSAs 3–4 under the x-directional loadings. It is noted that the impedance signatures of the CSA sensors showed clear and sensitive trends as compared to the existing smart aggregate sensors [25,30]. Moreover, the clear signals (see Figure 15a,b) pointed out that there were no effects of noise on the measured signatures.

#### 4.3.2. Experimental Impedance Features of CSAs

**Figure 16:**
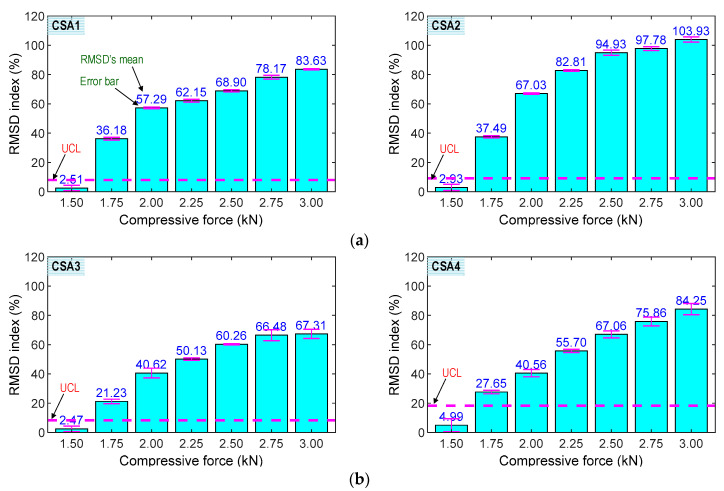
RMSD indices of CSAs under compression. (**a**) z-directional loading. (**b**) x-directional loading.

Figure 16 shows the RMSD indices of the impedance signatures of the CSA sample under compression forces P1–P7: (1) CSAs 1–2 under the z-directional loading (see Figure 16a) and (2) CSAs 3–4 under the x-directional loading (see Figure 16b). The upper control limit UCLs were calculated using the impedance signals at the applied forces P1 (i.e., the baseline). The error bars were also computed for each loading case. As shown in the figure, the RMSD indices were negligible in the intact case (P1) for all sensors, but they were increased and beyond the UCLs under the other cases (P2–P7), suggesting that the variations of the compressive forces were successfully monitored using the CSAs. The small error bars also indicate that the impedance signals were relatively stable. The CSAs under z-directional loading showed relatively higher sensitivity to applied force variation than those under x-directional loading.

**Figure 17:**
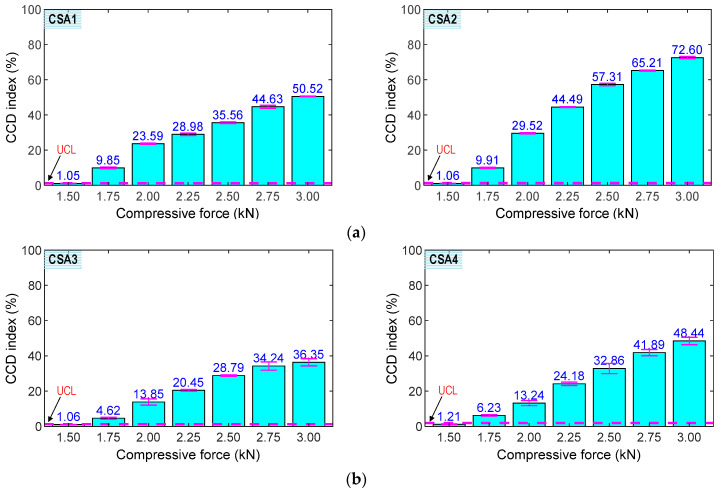
CCD indices of CSAs under compression. (**a**) z-directional loading. (**b**) x-directional loading.

Figure 17 shows the CCD indices of the impedance signatures of the CSA samples under applied forces P1–P7: (1) CSAs 1–2 under the z-directional loading (see Figure 17a) and (2) CSAs 3–4 under the x-directional loading (see Figure 17b). The upper control limit UCLs were computed using the impedances at the baseline P1. The error bars were calculated for each loading case. As observed in the figure, the CCD indices were ignorable under P1 for all sensors, but they were increased and beyond the UCLs under cases (P2–P7), suggesting that the alteration of the applied forces was successfully detected using the CSAs. The small error bars also indicate that the impedance signals were relatively stable. The CSAs under the z-directional loadings had relatively higher sensitivity to the force variation than those under the x-directional loadings. Furthermore, there were differences in the CCD magnitudes of CSA 1 compared to CSA 2 under the z-directional loadings and the CCD values of CSA 3 compared to CSA 4 under the x-directional loadings. These differences could be induced by the effects of the sensor fabrication, the bonding condition, and the compressive testing setup.

#### 4.3.3. Correlation between Impedance Features and Stresses

**Figure 18:**
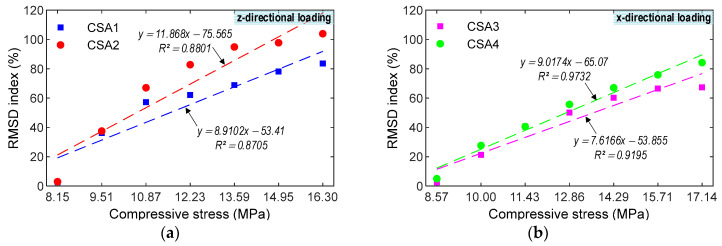
Experimental correlation estimation between RMSD index and compressive stress. (**a**) z-directional loading. (**b**) x-directional loading.

**Figure 19:**
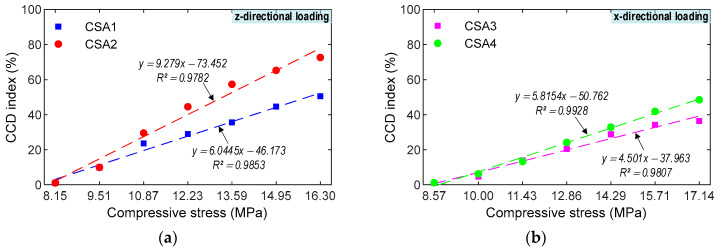
Experimental correlation estimation between CCD index and compressive stress. (**a**) z-directional loading. (**b**) x-directional loading.

The correlations were examined between the impedance features and stresses under the compressive loadings, as shown in Figure 18 and Figure 19. Furthermore, the correlations obtained from the CSA under the z-directional loadings were compared to the CSA under the x-directional ones to show a better one for impedance-based monitoring. Figure 18 shows the relationship between the RMSD index and stress. For the CSA under the z-directional loadings, *R*^2^ were 0.8705 (CSA 1) and 0.8801 (CSA 2). For the CSA under the x-directional loadings, *R*^2^ were 0.9195 (CSA 3) and 0.9732 (CSA 4). The CSAs under the x--directional loadings displayed a better fit than those under the z-directional loadings. Figure 19 shows the relationship between the CCD index and stress. For the CSA under the z-directional loadings, *R*^2^ were 0.9853 (CSA 1) and 0.9782 (CSA 2). For the CSA under the x-directional loadings, *R*^2^ were 0.9807 (CSA 3) and 0.9928 (CSA 4). The CSAs under the x-directional loading had better linear fitting effects than those under the z-directional loading. As observed in Figure 18 and Figure 19, the statistic indicators (i.e., RMSD and CCD) can be used to quantitatively monitor the stress in the CSA induced by the compression force. Furthermore, the CSAs under x-directional loading performed better impedance monitoring than the CSAs under z-directional loading.

#### 4.3.4. Discussion on CSA Sensor’s Feasibility

From the experimental analyses, the following three observations were made to verify the feasibility of the CSA sensor: (1) the effective frequency range of the CSA for impedance monitoring was pre-determined from 10 kHz to 40 kHz; (2) the effect of loading direction caused slight differences in the impedance features (i.e., RMSD and CCD indices) of the CSA; (3) the impedance features measured by the CSA were relatively consistent and sensitive to the change in compressive loadings; and (4) the correlation between the impedance signatures and stresses could be used to quantitatively monitor stress change in the CSA-embedded concrete structure. It is also noted that more CSA samples should be tested in the future to determine empirical formulas on impedance signatures’ compressive forces, which would be reliable enough for impedance-based stress monitoring in real structures.

For practical implementation, the CSA sensor could be installed in the target concrete structures to monitor: (1) concrete strength development [18,19], compressive stress, and inner damage occurring in concrete samples (e.g., standard cylinder or cube) under compression [20,31]; (2) prestress forces and internal tensile damage in prestressed concrete anchorage zone under prestressing forces [21,32]; and (3) flexural damage in a reinforced concrete beam [33].

## 5. Concluding Remarks

A new capsule-like smart aggregate (CSA) was developed and verified for impedance-based monitoring in a pre-determined frequency range of less than 100 kHz. The pros and cons of the existing smart aggregate sensors were discussed to define the requirement for the improved CSA model. The conceptual design and the impedance measurement model of the CSA sensor were demonstrated for concrete damage monitoring. In the model, the interaction between the CSA and the monitored structure was considered the 2-degrees of freedom (2-DOF) impedance system. The mechanical and impedance responses of the CSA during concrete strength development and under compressive loading conditions were figured out. Next, the prototype of the CSA was designed for impedance-based monitoring in concrete structure. The local dynamic properties of the CSA were numerically simulated to obtain impedance signatures in pre-determined frequency bands. Numerical and experimental impedance analyses were performed for the CSA to investigate its sensitivity under compressive loadings. The alterations in the impedance signals of the CSA induced by the compressive loadings were analyzed to assess the effect of loading directions on the performance of the CSA. The correlations between statistical impedance features and compressive stresses were also made to examine the feasibility of the CSA for stress quantification.

From the numerical and experimental investigations on the CSA sensor, at least four concluding remarks can be made as follows. Firstly, the feasibility of the CSA for low-frequency impedance monitoring (less than 100 kHz) was successfully evaluated. Secondly, the sensitive frequency range of the CSA was pre-determined in the range of 10–40 kHz via numerical and experimental analyses. Thirdly, the impedance features of the CSA were increased under a series of compressive loadings. There exist linear relationships between the statistical impedance features and compressive stresses of the CSA, suggesting that the CSA has the potential for axial force/stress variation monitoring and concrete damage detection in real concrete structures. Finally, the CSAs under x-directional loading showed a better performance for impedance monitoring than the CSAs under z-directional loading, suggesting that the CSA should be localized in the case of the vibrating plate parallel to the applied force.

## Figures and Tables

**Table 1:** Material properties of aluminum, bonding layer, and PZT patch.

Properties	Aluminum 6061-T6	PZT 5A	Bonding Layer Epoxy	Bonding Layer Super Glue
Young’s modulus, *E* (GPa)	68.9	62.1	0.75	5
Poisson’s ratio, *ν*	0.33	0.35	0.30	0.38
Mass density, *ρ* (kg/m^3^)	2700	7750	1090	1700
Damping loss factor, *η*	0.02	0.0125	0.02	
Yield strength, σ_y_ (MPa)	241			
Compressive strength, σ_c_ (MPa)			32.3	
Dielectric constant, *ε^T^*^33^ (F/m)		1.53 × 10^−8^		
Coupling constant, *d*_31_ (m/V)		−1.71 × 10^−10^		
Dielectric loss factor, *δ*		0.015		

## Data Availability

Data available on reasonable request from the corresponding author.
